# Patient benefit of dog-assisted interventions in health care: a systematic review

**DOI:** 10.1186/s12906-017-1844-7

**Published:** 2017-07-10

**Authors:** Martina Lundqvist, Per Carlsson, Rune Sjödahl, Elvar Theodorsson, Lars-Åke Levin

**Affiliations:** 1 Department of Medical and Health Sciences, Division of Health Care Analysis, Linköping University, 581 83, Linköping, Sweden; 2 Department of Clinical and Experimental Medicine, Linkoping University, Linköping, Sweden; 3 Department of Clinical Chemistry and Department of Clinical and Experimental Medicine, Linköping University, Linköping, Sweden

**Keywords:** Animal-assisted therapy, Cost-benefit analysis, Dogs, Dog-assisted activity, Dog-assisted intervention, Dog-assisted support, Dog-assisted therapy, Outcome assessment (health care), Quality of life, Treatment outcome

## Abstract

**Background:**

Dogs are the most common companion animal, and therefore not surprisingly a popular choice for animal-assisted interventions. Dog-assisted interventions are increasingly used in healthcare. The aim of the review was to conduct a systematic literature review of quantitative studies on dog-assisted interventions in healthcare, with the intention of assessing the effects and cost-effectiveness of the interventions for different categories of patients.

**Methods:**

A **s**ystematic review of the scientific literature reporting results of studies in healthcare, nursing home or home care settings, was conducted. The inclusion criteria applied for this review were: quantitative studies, inclusion of at least 20 study subjects, existence of a control and performed in healthcare settings including nursing homes and home care. The electronic databases PubMed, AMED, CINAHL and Scopus were searched from their inception date through January 2017, for published articles from peer-reviewed journals with full text in English.

**Results:**

Eighteen studies that fulfilled the inclusion criteria, and were judged to be of at least moderate quality, were included in the analysis. Three of them showed no effect. Fifteen showed at least one significant positive effect but in most studied outcome measures there was no significant treatment effect. Dog-assisted therapy had the greatest potential in treatment of psychiatric disorders among both young and adult patients. Dog-assisted activities had some positive effects on health, wellbeing, depression and quality of life for patients with severe cognitive disorders. Dog-assisted support had positive effects on stress and mood.

**Conclusions:**

The overall assessment of the included studies indicates minor to moderate effects of dog-assisted therapy in psychiatric conditions, as well as for dog-assisted activities in cognitive disorders and for dog-assisted support in different types of medical interventions. However, the majority of studied outcome measures showed no significant effect.

**Electronic supplementary material:**

The online version of this article (doi:10.1186/s12906-017-1844-7) contains supplementary material, which is available to authorized users.

## Background

Interaction with animals has been a favorite human pursuit since the dawn of history. Numerous studies have reported that animals exert favorable effects on psychological, physiological and social aspects of human wellbeing [1]. The increasing use of animals in health and social care is therefore not surprising.

Animal-Assisted Interventions (AAI) are more or less goal oriented and structured interventions that intentionally incorporate animals in health, education and human service for the purpose of therapeutic gains and improved health and wellness [2]. AAI usually consists of three sub-categories; Animal-Assisted Therapy (AAT), Animal-Assisted Education (AAE) and Animal-Assisted Activities (AAA). These concepts are defined in a slightly different way by various organizations. According to the International Association of Human-Animal Interaction Organizations (IAHAIO), AAT is a goal oriented, planned and structured therapeutic intervention directed and/or delivered by health, education and human service professionals, and intervention progress is measured and included in professional documentation [3]. According to the American Veterinary Medical Association (AVMA), the definition AAT should also be an integral part of the treatment process [4]. Most definitions of AAA differ from AAT in several aspects; the absence of specific treatment goals, delivery by volunteers, spontaneous visit content and no obligation to document. According to the IAHAIO definition, AAA is a “planned and goal oriented informal interaction and visitation conducted by the human-animal team for motivational, educational and recreational purposes. Human-animal teams must have received at least introductory training, preparation and assessment to participate in informal visitations.” [3]. Another difference between AAT and AAA seems to be the purpose, which is more focused on wellbeing in AAA and on health improvement in AAT. We also understand that AAA could be provided by different degrees of integration with a formal treatment process. However, this leads us to conclude that there are no established definitions and that there is no clear division between AAT and AAA.

Dogs are the most common companion animal and therefore not surprisingly a popular choice for AAI [1]. They are keen observers of human reactions through their exceptional ability to read signs of will and emotion from human faces [5]. They also exhibit a behavior that humans interpret as happy, friendly and affectionate which makes them suitable to be used in interventions with a therapeutic aim [1]. This review is therefore delimited to interventions with dogs as the assisting animal, transposing the term AAT to the corresponding Dog-Assisted Therapy (DAT) and AAA to Dog-Assisted Activity (DAA).

**Fig. 1:**
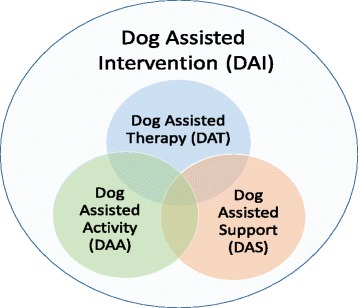
Dog-assisted intervention (DAI) comprises dog-assisted therapy (DAT), dog-assisted activity (DAA) or dog-assisted support (DAS)

In our review we have identified another relevant subcategory to Dog-Assisted Interventions (DAI), namely Dog-Assisted Support (DAS). DAS is delivered by a trained health care professional or a volunteer, within the scope of the professionals’ practice, in order to have alleviating or distracting effects during short term diagnostic or therapeutic interventions. In our opinion, DAS deserves to be a separate category since DAS in contrast to DAA has a specific diagnostic or therapeutic intention, especially in reducing anxiety and stress both in diagnostic and therapeutic contexts, but compared to DAT, DAS has no specific therapeutic goal. Rather, DAS focuses on reduced anxiety and quality of life with indirect effect to facilitate a regular treatment process. Figure 1 illustrates our identified categories.

Previous reviews of DAI studies [6–10] have focused exclusively on single conditions or on specific populations. In this review we have included various conditions without any limitation of the characteristics of the population, except for sample size.

## Aim

The aim of the review was to conduct a systematic literature review of quantitative studies on dog-assisted interventions in healthcare, with the intention of assessing the effects and cost-effectiveness of the interventions for different categories of patients.

## Method

### Search methods for identification of studies

Studies were identified by searching the electronic databases PubMed, AMED, CINAHL and Scopus from their inception date through January 2017. Some of the included studies were identified outside standard database searches (e.g. hand searching reference lists from included articles and systematic reviews).

We searched for various terms of DAI and outcomes. The search was conducted as presented in Table 1.

**Table 1:** Search strategy

*Dog OR canine*	
AND	
Animal-assist* OR Dog-assist* OR Pet-assist* OR Canine-assist* OR animal-therap* OR dog-therap* OR pet-therap* OR canine-therap* OR “animal visitation” OR “dog visitation” OR “pet visitation” OR “canine visitation” OR animal-physiotherap* OR dog-physiotherap* OR pet-physiotherap* OR canine-physiotherap* OR therapy-dog OR visiting-dog	
AND	
*Effect OR effectiveness OR benefit OR quality of life OR economics OR cost-effectiveness OR ethics OR outcome*	

### Selection of studies

The inclusion criteria applied in the review were determined before the initial literature search. The first four criteria were a consequence of the scope of the study. The last three criteria were determined by the research group as basic quality criteria to be fulfilled. The inclusion criteria;

- Studies of DAI performed in healthcare settings including nursing homes and home care.
- Studies collecting primary data.
- Quantitative studies (i.e. measuring outcomes with numerical scales).
- Separate result presentation of effects.
- At least 20 study subjects.
- Existence of a control.
- Published articles from peer-reviewed journals with full text in English.

One of the authors (ML) conducted the initial search in May 2016, as well as a second search in September 2016 and a third search in January 2017. All the titles and abstracts from the identified studies were examined to determine the relevance of the articles. If the title and abstract met with the inclusion criteria the study passed on to the next stage of the review process. Studies with insufficient information in title and abstract were also included in the next stage of the review process. The studies that remained after the initial selection, were read in full to confirm eligibility and determine quality.

### Review of quality

Pairs of authors independently read the articles in full to assess eligibility and to determine the quality. Traditional criteria for judgment of scientific quality were used, including; adequate control group(s), control of confounders, randomization, adequately described experimental design, and relevant measured outcome variables [11, 12]. The quality of the studies was rated as high, moderate or low. A study determined as being of high quality had to fulfill all the above mentioned criteria and a study determined as being of moderate quality had to fulfill most of the above mentioned criteria. When a study was determined as being of low quality it failed to fulfill several of the above mentioned criteria or had major shortcomings in some of the criteria. Studies rated as moderate or high were included in the analysis. Studies rated as low quality were excluded, but are presented with a comment in an additional file (see Additional file 1). For the included studies the following data was extracted and gathered into a structured table; reference, characteristics of patients (age, gender, condition), type of study, type of intervention, study period, sample size, outcomes, author conclusion, and scientific quality rating.

### Categorization

To establish in what context DAIs have effect we categorized the interventions as therapeutic (DAT), activating (DAA) or supportive (DAS) intervention, based on the criteria presented in Table 2.

**Table 2:** Criteria for categorization of interventions

	DAT	DAA	DAS
Intervention			
Aim of action	Goal orientated	No specific goals	No specific goals
Content of activity	Structured	Spontaneous	Spontaneous
Amount of activity	Multiple sessions	Multiple sessions	Single session
Treatment integration	High	Low	High
Dog handler/therapist	Educated	Volunteer	Volunteer
	Professional	Not professional	Semi-professional
Effect/Focus	Health	Well-being	Distraction Stress reduction

## Results

### Results of the search

**Fig. 2:**
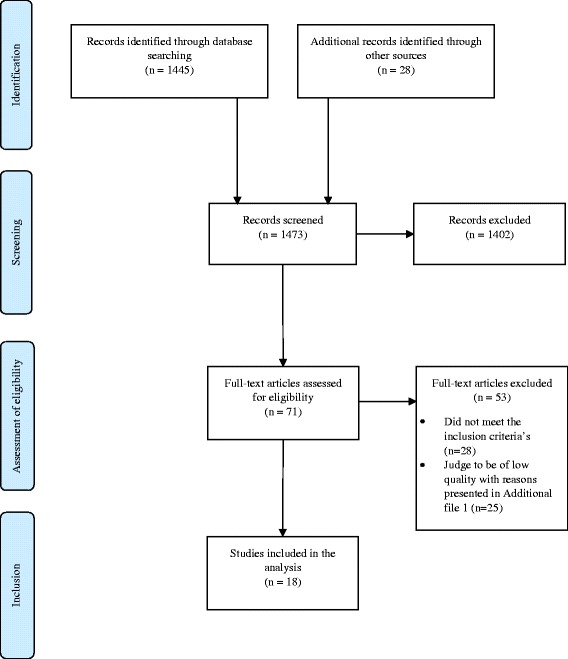
Flow chart of the work process. PRISMA 2009 Flow Diagram [58]

The result of the search is illustrated in the flow chart in Fig. 2. It resulted in 1445 unique articles (after duplicate removal). Another 28 studies were included through other sources. Based on the information in the title and the abstract 1402 articles did not meet the eligibility criteria and were therefore excluded. The main reason for early exclusion was that the article did not scope DAI. Additionally, 53 articles were excluded after reading the full text. Of these 53 articles, 28 were excluded since they did not meet the inclusion criteria and 25 were excluded due to low quality [13–58] (see Additional file 1). Finally, 18 studies that fulfilled the inclusion criteria, and were judged to be of at least moderate quality, were included in the final analysis. None of the studies were considered to be of high quality. A summary of the 18 studies included in the final analysis is presented in Tables 3 and 4.

**Table 3:** Summary of studies included in the review

First author (year)	Patients				Study design	Sessions			Intervention	Control
	N	Mean age (years)	Gender (% male)	Target group		Duration (weeks)	Number	Length (minutes)		
Lutwack-Bloom (2005) [38]	68	69.9	41.8	Residents living in long-term care setting	Pre-post	24	72	15–20	Visit from a therapy dog	Visit without dog
Majic (2013) [39]	54	81.7	29.6	Residents with dementia	Pre-post	10	10	45	Dog assisted therapy	Regular therapy
Travers (2013) [40]	55	85	21.8	Residents with dementia	Randomized control	11	22	40–50	Dog assisted therapy	Regular therapy
Bono (2015) [41]	24	82.1	33.3	Non-hospitalized patients with low-mild Alzheimer’s disease	Randomized control	32	16	60	Dog assisted therapy	No active intervention
Friedmann (2015) [42]	40	80.7	27.5	Assisted living residents with cognitive impairment/Dementia	Randomized control	12	24	60–90	Dog assisted therapy	Regular therapy
Olsen (2016) [43]	51	Experimental 82.9 Control 84.1	37.3	Residents with dementia	Randomized control	12	24	30	Dog assisted therapy	Regular therapy
Thodberg (2016) [44]	100	85.5	31	Nursing home residents/Dementia	Randomized control	6	12	10	Visit from a therapy dog	Visits from a person bringing a robot seal or soft toy cat
Schuck (2015) [45]	24	Experimental 7.99 Control 7.81	83.3	Children with ADHD	Randomized control	12	24	120–150	Dog assisted therapy	Regular therapy
Stefanini (2015) [46]	34	15.9	52.9	Children with mental disorders	Randomized control	12	12	45	Dog assisted therapy	Regular therapy
Calvo (2016) [47]	22	47.8	70.8	Patients with schizophrenia	Randomized control	24	40	60	Dog assisted therapy	Regular therapy
Stefanini (2016) [48]	40	Experimental 15.2 Control 16.4	45	Children with mental disorders	Randomized control	12	10	45	Dog assisted therapy	Regular therapy
Nagengast (1997) [49]	23	4.7	39.1	Children undergoing physical examination	Within-subject, time series design	⎼	2	10	Dog present during physical examination	Physical examination without dog
Hansen (1999) [50]	34	Experimental 4.1 Control 3.5	41.2	Children undergoing physical examination	Randomized control	⎼	1	⎼	Dog present during physical examination	Physical examination without dog
Havener (2001) [51]	40	8.36	42.5	Children undergoing dental procedures	Randomized control	⎼	1	⎼	Dog present during dental procedure	Dental procedure without dog
Johnson (2008) [52]	30	Dog visits 61 Human visits 59 Reading 58	30	Patients undergoing non-palliative radiation therapy	Randomized control	4	12	15	Visit from a therapy dog	Visit without dog or reading sessions
Vagnoli (2015) [53]	50	Experimental 7.1 Control 7.38	48	Children undergoing venipuncture	Randomized control	⎼	1	10–15	Dog present during venipuncture	Venipuncture without dog
Krause-Parello (2016) [54]	28	82.9	42.9	Older adults	Crossover	⎼	2	60	Visit from a therapy dog	Visit without dog
Harper (2015) [55]	72	Experimental 67 Control 66	41.7	Patients undergone total joint arthroplasty	Randomized control	⎼	3	15	Physical therapy with a therapy dog	Physical therapy

**Table 4:** Summary of outcomes from studies included in the review

First author (year)	Condition	Intervention	Outcomes of DAI	Author’s conclusion
Lutwack-Bloom (2005) [38]	Cognitive disorders	Activating	⎼Geriatric Depression Scale (GDS) ↑Profile of Mood States (POMS)	The results suggest that the findings indicated that the intervention works better with general mood disorders than with depression alone.
Majic (2013) [39]	Cognitive disorders	Activating	⎼Dementia Mood Assessment Scale (DMAS) ⎼Mini-Mental State Examination (MMSE) ⎼Cohen-Mansfield Agitation Inventory (CMAI)	AAT is a promising option for the treatment of agitation/aggression and depression in patients with dementia.
Travers (2013) [40]	Cognitive disorders	Activating	↑↓Quality of Life-Alzheimer’s Disease (QOL-AD)^a^ ⎼The MOS-Item short form health survey (SF-36) ⎼Geriatric Depression Scale Short Form (GDS-SF) ↑Multidimensional Observational Scale for Elderly Subjects (MOSES)^b^	The authors conclude that the study provides some evidence that dog-assisted therapy may be beneficial for some residents of aged care facilities with dementia.
Bono (2015) [41]	Cognitive disorders	Activating	↑Barthel Index ↑Alzheimer Disease Assessment Scale (ADAS) ↓Cornell Scale for Depression in Dementia (CSDD)	The study confirms the feasibility of AAT with dogs in low-mild Alzheimer’s disease
Friedmann (2015) [42]	Cognitive disorders	Activating	⎼Barthel Index - Physical function ↑Cornell Scale for Depression in Dementia (CSDD) ⎼AES – Emotional function ⎼Cohen-Mansfield Agitation Inventory (CMAI)	Evidence supports that the PAL program helps preserve/enhance function of AL residents with CI.
Olsen (2016) [43]	Cognitive disorders	Activating	↑Cornell Scale for Depression in Dementia (CSDD) ⎼Brief Agitation Rating Scale (BARS) ↑Quality of Life in Late-stage Dementia (QUALID)^c^	Animal-assisted activities may have a positive effect on depression and QoL in older people with dementia.
Thodberg (2016) [44]	Cognitive disorders	Activating	⎼Mini-Mental State Examination (MMSE) ⎼Gottfried-Brane-Steen scale (GBS) ⎼Geriatric Depression Scale (GDS) ⎼Sleep data ⎼Body Mass Index (BMI)	Visit type did not affect the long-term mental state of the patients. The relationship between sleep duration and dog-accompanied visits remains to be explored.
Schuck (2015) [45]	Psychiatric disorders	Therapeutic	↑ADHD-Rating Scale-Fourth edition (ADHD-RS-IV) ↑Social Skills Improvement Systems-Rating Scales (SSIS-RS) ↑Social Competence Inventory (SCI)	Results suggest that CAI offers a novel therapeutic strategy that may enhance cognitive-behavioral interventions for children with ADHD.
Stefanini (2015) [46]	Psychiatric disorders	Therapeutic	↑Children Global Assessment Scale (CGAS) ↑Format of hospital care scale ↑Ordinary school attendance scale ↑Patients behaviors during AAT	Our results verify that AAT can have significant positive effects on therapeutic progress and the recovery process.
Calvo (2016) [47]	Psychiatric disorders	Therapeutic	⎼Positive and Negative Syndrome Scale (PANSS) ⎼EQ-5D – Quality of life ↑Adherence to Treatment ↑ Decrease in saliva cortisol	The results suggest that AAT could be a useful adjunct to conventional psychosocial rehabilitation for people with schizophrenia.
Stefanini (2016) [48]	Psychiatric disorders	Therapeutic	↑Children Global Assessment Scale (CGAS) ↑Youth Self Report (YSR) ↑Patients behaviors during AAT^d^	The effects of AAT in reducing emotional and behavioral symptoms and increasing global competence and psychological functioning were substantiated.
Nagengast (1997) [49]	Stress and mood	Supportive	↑Systolic blood pressure ⎼Diastolic blood pressure ↑Mean arterial blood pressure ↑Heart rate ⎼Peripheral skin temperature ↑Observation Scale of Behavioral Distress (OSBD)	The findings support the use of a companion animal in reducing stress experienced by children during physical examination.
Hansen (1999) [50]	Stress and mood	Supportive	⎼Systolic blood pressure ⎼Diastolic blood pressure ⎼Mean arterial blood pressure ⎼Heart rate ⎼Peripheral skin temperature ↑Observation Scale of Behavioral Distress (OSBD)	Companion animals may be useful in a variety of health care settings to decrease procedure-induced distress in children.
Havener (2001) [51]	Stress and mood	Supportive	⎼Peripheral skin temperature ⎼Observation Scale of Behavioral Distress (OSBD)	The authors conclude that further research using a larger sample should be done to determinate the effect of a companion animal with children for whom the dental visit is most stressful.
Johnson (2008) [52]	Stress and mood	Activity	⎼Profile of Mood states (POMS) ⎼Self-perceived health questionnaire ⎼Orientation to Life Questionnaire (OTLQ)	The study warrants replication with a larger sample to determine applicability of animal-assisted activity in cancer patients with radiation therapy.
Vagnoli (2015) [53]	Stress and mood	Supportive	↑Observation Scale of Behavioral Distress (OSBD) ⎼The Wong-Baker Scale (Face scale) ⎼Visual analog scale (VAS) - pain ⎼State Trait Anxiety Inventory (STAI) ↑Serum cortisol plasma	The presence of dogs during venipuncture reduces distress in children and improves physical, social, emotional, and cognitive functioning.
Krause-Parello (2016) [54]	Stress and mood	Activity	↑Systolic blood pressure ⎼Diastolic blood pressure ↑Heart rate	Study findings supported that pet therapy significant decreased blood pressure and heart rate.
Harper (2015) [55]	Pain	Supportive	↑Visual analog scale (VAS) - pain Hospital Consumer Assessment of Healthcare Providers and Systems survey (HCAHPS)^e^	The use of therapy dogs has a positive effect on patients’ pain level and satisfaction with hospital stay after total joint replacement.

↑ Statistical significant improvement for the DAI compared to the alternative

↓ Statistical significant impairment for the DAI compared to the alternative

⎼ No statistical significant change between the alternatives

^a^Significant improvement in one facility and significant impairment in another. The impairment may have been effected by an outbreak of gastroenteritis

^b^Patients with higher (worse) baseline scores

^c^Patients with severe dementia

^d^§Indicating improvement in motivation, relationship, socialization, cure and affects

^e^Patients in the intervention group had higher proportions of top box scores in categories of nursing communication and pain management

### Included studies

The included studies were published in the time period 1997–2016. Countries of the corresponding authors that were represented were USA (8 studies), Italy (4 studies), Australia, Columbia, Denmark, Germany, Norway, Spain (1 study each).

Seventy-eight percent of the studies were randomized controlled trials. Two studies were cohort studies with a pre-post intervention. One study adopted a within-subject, time series design and one used a crossover design.

### Studied patients

The number of patients included in the studies varied, between 23 and 100. Eleven studies had 20–40, four had 41–60, two had 61–80, and one had 81–100 patients (Table 3). In 11 of the 18 studies the patients were adults. The remaining studies included children and adolescents. The gender distributions favored women, 14 of the 18 studies had >50% women.

### Studied disorders

Studies on *cognitive disorders* dominated the selected studies [38–44]. In four studies the patient conditions were *psychiatric disorders,* of which three included children or adolescents with psychiatric disorders and one included patients cared for in institutions due to psychiatric disorders [45–48]. Six studies examined *stress and mood* [49–54]. Target symptoms and diseases were children undergoing physical examination, children undergoing dental procedure, children undergoing venipuncture, patients with cancer and older adults. In one study the intervention was preformed to reduce *pain* for patients undergoing total knee joint arthroplasty [55].

### Studied controls

In all studies expect two, the control treatment was a visit, series of visits, a therapy session or series of therapy sessions without a dog. In the study by Thodberg et al. the controls were either a visit from a person bringing a robot seal or a visit from a person bringing a soft-toy cat [44]. In the study by Bono et al. the control group received no active intervention [41].

### Outcomes

The studies included a variety of outcome measures and instruments (Table 4), physiological parameters e.g. blood pressure, heart rate, cortisol in saliva, skin temperature; disease specific measures e.g. dementia mood assessment scale, geriatric depression scale, Cohen-Mansfield agitation inventory; general functional measures e.g. observation scale of behavioral distress, profile of mood states, self-perceived health questionnaire, orientation to life questionnaire; generic health related quality of life measures e.g. EQ-5D, quality of life in late-stage dementia; and other measurements including e.g. sleep data, body mass index, and ordinary school attendance. In the column ‘Outcomes of DAI’ (Table 4) we have summarized the outcome changes for the different studies, based on statistical significance.

Table 5 shows the number of studies with at least one statistically significant positive outcome measure divided by patient condition and intervention category.

**Table 5:** Number of studies divided into condition, type of intervention, and the presence of positive outcome

Condition	Therapeutic intervention		Activating intervention		Supportive intervention	
	At least one significant positive outcome					
	Yes	No	Yes	No	Yes	No
Cognitive disorder			6	1		
Psychiatric disorder	4					
Stress and mood			1	1	3	1
Pain					1	

#### Cognitive disorders

Seven trials studied cognitive disorders and were all categorized as DAA [38–44]. They differed in terms of patient population severity, which varied from mild cognitive impairment to severe dementia. In all studies multiple sessions were used during periods varying in length from 6 to 32 weeks. The number of sessions varied between 12 and 72, and the length of sessions were 10–90 min.

The studies also differed regarding control group treatments. In Friedman et al., Thodberg et al. and Lutwack et al. the control group participants were given another structured intervention [38, 42, 44]. In Majic et al., Olsen et al. and Travers et al. the control group was treated as usual [39, 40, 43]. In the study conducted by Bono et al. the control group received no active intervention [41].

Two of the studies showed some decrease in depression specific instruments [40, 42]. Travers et al. showed that patients with worse baseline depression scores in the DAA group, significantly improved depression scores in comparison to the control group. They also found significant improvements in quality of life in one of the facilities studied, but these results were confounded by an outbreak of gastroenteritis with subsequent significant decrease in QoL in another unit [40]. In the Friedman et al. study, depression decreased significantly in the DAA group after three months but not in the control group [42]. In the study by Olsen et al. patients with severe dementia in the DAA group had an improved quality of life at follow up [43]. Bono et al. showed a significant difference in reduction of functional status between the DAA group and the control group after eight months. In the DAA group, the development of cognitive impairment also slowed up in comparison to the control group [41]. In Thodberg et al. the sessions lasted only 10 min and the control group were activated with a robot seal or a soft toy cat. They found no effect in measures of depression [44]. Also in Lutwack et al. the patients only received short sessions, the results showed no effect in measures of depression but a significant improvement in mood for those receiving visits from a therapy dog [38].

Concerning cognitive disorder, we conclude that the included studies differed in terms of severity of patient conditions, and in particular in the characteristics of DAA and the activity offered to the control group. Given these differences, treatment of cognitive disorders in a nursing home setting may result in some positive effects on health and wellbeing, most likely on depression and on quality of life for patients with severe dementia.

#### Psychiatric disorders

The four trials that studied psychiatric disorders were all categorized as DAT, and were randomized in a pre-post experimental design. Three of the included studies investigated patients in child and adolescent psychiatry [45, 46, 48], and one studied patients from adult psychiatry [47]. All of the studies comprised 12 week DAT programs in different settings except for the study conducted by Calvo et al., where the intervention lasted 24 weeks [47].

In both studies conducted by Stefanini et al. DAT was compared with standard treatment in children and adolescents admitted to a psychiatry unit for acute mental disorder [46, 48], while Schuck et al. compared cognitive-behavioral intervention delivered with or without DAT in children with ADHD [45]. In the study by Calvo et al. [47] of adult inpatients with schizophrenia, DAT was assessed as an adjunct to, and in comparison with, conventional psychosocial rehabilitation.

All three studies of young patients with psychiatric disorder showed that DAT resulted in significant improvements on different psychometric scales and measures [45, 46, 48]. The two DAT studies, studying young patients with acute mental disorders, found improvements in global functioning, school attendance, as well as self-reported emotional-behavioral symptoms [46, 48]. DAT in ADHD children resulted in a greater reduction in the severity of ADHD symptoms in comparison to cognitive-behavioral interventions without DAT [45]. Calvo et al. showed that after 24 weeks of rehabilitation the DAT group had no benefit on the Positive and Negative Syndrome Scale (PANSS), but in comparison with conventional rehabilitation, a significant reduction of negative symptomatology, a higher adherence to the program, and cortisol reduction were found after the DAT sessions [47].

In summary, all studies of DAT in psychiatric disorders showed significant reductions in symptoms, and higher program adherence.

#### Stress and mood

Seven trials studied stress and mood, five of them were categorized as DAS and two as DAA. Nagengast et al., Hansen et al. and Havener et al. examined effects of DAS on physiological and behavioral distress among children undergoing a physical examination or a dental procedure [50, 51]. Vagnoli et al. studied the effects of DAS in children undergoing venipuncture [53]. Johnson et al. conducted a study in a radiation oncology unit examining effects of DAA among patients undergoing non-palliative radiation therapy [52]. Krause-Parello et al. [54] investigated changes in older adult’s cardiovascular health before and after a DAA.

In the study by Nagengast et al. the children were exposed to two examinations, one with and one without a dog present [49]. In Hansen et al., Havener et al. and Vagnoli et al. the children underwent a procedure with or without the presence of a dog [50, 51, 53]. In the study by Johnson et al. two control groups were constructed, one receiving visits from a human person and one where the patients read magazines [52]. In the study by Krause-Parello et al. all patients received two visits; one with and one without a dog [54].

In four of the six studies, the populations studied were children and the number of sessions was limited to one [49–51, 53]. Both Nagengast et al. and Hansen et al. found lower stress levels during physical examination when a dog was present, compared to not present, measured with the Observation Scale of Behavioral Distress (OSBD) [49, 50]. In addition, Nagengast et al. reported a statistically significant decrease in mean arterial and systolic blood pressure, heart rate and behavioral distress in the presence of a dog [49]. Vagnoli et al. also reported significant lower stress levels measured with OSBD, when a dog was present, in children undergoing venipuncture [53]. They also found significant lower levels of serum cortisol plasma in the intervention group compared with the control group [53]. The study conducted by Havener et al., examining children undergoing a dental procedure, was very similar to the other studies but showed no significant effects [51]. The study conducted by Johnson et al. differed from the other studies. They investigated the effects of DAA during a four-week period, including 12 sessions on adults undergoing non-palliative radiation therapy. They found no effects of DAA [52]. The study by Krause-Parello et al. also differed from the other studies [54]. The study was conducted on older adult patients living at home who did not have any specific condition. The results implied a greater decrease in systolic blood pressure when visited by a dog compared to a human person. From the results they also predicted more of a decrease in heart rate during the DAA compared with the conventional intervention [54].

In summary, four out of six studies showed at least one significant positive effect. Taken together, these findings suggest that particular DAS may reduce stress and positively affect the mood.

#### Pain

Harper et al. studied the role of DAS in postoperative recovery in patients after total joint arthroplasty [55]. Patients in the intervention group received a 15-min visit from a dog before the patient underwent physical therapy over a three-day period. The control group underwent physical therapy without any changes to normal routine. Patients in the intervention group reported a significant reduction of pain measured with the visual analog scale (VAS) compared with the control group [55]. Like the other studies categorized as supportive interventions reporting positive effects, the study included few and short sessions.

## Discussion

Even though there is a growing number of studies reported on use of animals in healthcare, the evidence base appears weak, partly due to studies including a limited number of participants. Previous reviews of AAI studies have focused exclusively on a single condition or a specific population. This review was delimited to dogs used as the assisting animal in a health care setting but without any limitation of the characteristics of the population. The studies fulfilling our selection criteria included diverse conditions and outcome measures. The findings were not consistent, and studies of cost-effectiveness were lacking.

Substantial differences were observed in the manner in which DAI was applied. Three major categories of interventions were identified; those used for therapeutic purpose, those used for activating purpose and those used as support during a procedure. Even if we were unable to draw firm conclusions regarding specific applications from the studies included in the current review, some promising results were seen. DAT seemed to be most substantial in treatment of psychiatric disorders both among young and adult patients. DAA appeared to have some positive effects on health and wellbeing as well as an effect on depression and quality of life in severe cognitive disorders. We also found that DAS may have positive effects on stress and mood.

Previous systematic reviews of the scientific literature regarding the use of DAI in health care settings showed some evidence of patient benefit. However, many reviews included a mix of animals and only a handful focused exclusively on DAI. One review of therapy dogs for children with autism spectrum disorders, included four studies on DAI in health care. It concluded that the results were encouraging but further research with better designs and larger samples were needed [56]. Another review of AAT in treatment of similar patient material included fourteen studies with different kinds of animals. Seven of these studies involved dogs and showed positive outcomes. However, most studies included were connected with methodological weaknesses [8]. The evidence base seemed to be better for the use of DAI for residents of long-term care facilities. Cipriani et al. showed that 12 of 19 studies reported statistically significant outcomes [57]. Even though there is some systematic, and a substantial amount of anecdotal evidence for the effects of animals as interventions in health care, the evidence base seems to be relatively weak.

Overall, in all the included studies, DAIs were offered in combination with regular medical treatment, which means that the control group always received regular treatment. In general though, we found that the included studies were fraught with numerous obstacles and confounding factors. For example, lack of proper control group and lack of a proper control activity, were common. Also, most studies had not attempted to investigate the impact of the dog handler. Besides that, there were natural difficulties in randomization due to patient preferences, fears and possible allergies. Given all the challenges mentioned above, it was a matter of concern that the number of participants was generally too small to minimize the effects of the multitude of lack of control and confounding factors involved. It is possible, and even likely, that matters of suboptimal study designs may explain some of the observed differences in the effects of DAI. It is also likely that the limited number of subjects included in most of the studies explained the non-statistically significant effects (type II statistical error). It is also possible that the instruments used in the studies for measuring treatment effects did not optimally measure the effect of all important aspects of increased wellbeing. Studies of DAI is evidently challenging regarding research design, since none of the studies we selected fulfilled our criteria of “high quality”.

Only 18 studies were included in the review as a primary basis for our conclusions. This limited number may be due to the strict criteria applied by us, to poor study designs, or both. One weakness with our review may be that we excluded studies with less than 20 participants. It is possible that there exist studies based on small samples that could be judged to have at least moderate quality. There is also a possibility that we have wrongly classified one or several studies, but that would hardly affect our conclusion. Another weakness of a review linked to inclusion criteria, is the limitation of including only published articles from peer-reviewed journals with full text in English. This is a common delimitation due to a lack of resources, but of course a weakness.

## Conclusion

The overall assessment of the included studies indicates minor to moderate effects of dog-assisted therapy in psychiatric conditions, as well as for dog-assisted activities in cognitive disorders and for dog-assisted support in different types of medical interventions. However, the majority of studied outcome measures showed no significant effect.

## Additional file

Additional file 1: Excluded studies due to low quality with reasons for exclusions. (DOCX 49 kb)

## Abbreviations

AAA: Animal-assisted activity
AAE: Animal-assisted education
AAI: Animal-assisted intervention
AAT: Animal-assisted therapy
ADAS: Alzheimer Disease Assessment Scale
ADHD-RS-IV: ADHD-Rating Scale-Fourth edition
BARS: Brief Agitation Rating Scale
BMI: Body Mass Index
C-GAS: Children Global Assessment Scale
CMAI: Cohen-Mansfield Agitation Inventory
CSDD: Cornell Scale for Depression in Dementia
DAA: Dog-assisted activity
DAI: Dog-assisted intervention
DAS: Dog-assisted support
DAT: Dog-assisted therapy
DMAS: Dementia Mood Assessment Scale
GBS: Gottfried-Brane-Steen scale
GDS: Geriatric Depression Scale
HCAHPS: Hospital Consumer Assessment of Healthcare Providers and Systems survey
MMSE: Mini-Mental State Examination
MOSES: Multidimensional Observational Scale for Elderly Subjects
OSBD: Observation Scale of Behavioral Distress
OTLQ: Orientation to Life Questionnaire
PANSS: Positive and Negative Syndrome Scale
POMS: Profile of Mood States
QOL-AD: Quality of Life-Alzheimer’s Disease
QUALID: Quality of Life in Late-stage Dementia
SCI: Social Competence Inventory
SSIS-RS: Social Skills Improvement Systems-Rating Scales
STAI: State Trait Anxiety Inventory
VAS: Visual Analog Scale
YSR: Youth Self Report
