# Supplementary material for: Patient benefit of dog-assisted interventions in health care: a systematic review
Source: BMC Complement Altern Med. 2017 Jul 10;17:358. doi: 10.1186/s12906-017-1844-7 (PMC5504801; doi:10.1186/s12906-017-1844-7)
Supplement: Additional file 1: — Excluded studies due to low quality with reasons for exclusions. (DOCX 49 kb) [file 12906_2017_1844_MOESM1_ESM.docx]

# Excluded studies due to low quality with reasons for exclusion

| Exclusion no. | First author (year) | Title | Reason for quality rated low |
| --- | --- | --- | --- |
| 1 | Crowley-Robinson (1996) [34] | A long-term study of elderly people in nursing homes with visiting and resident dogs | 1, 4 |
| 2 | Zisselman (1996) [35] | A Pet Therapy Intervention With Geriatric Psychiatry Inpatients | 2, 4, 6 |
| 3 | Barker (1998) [36] | The effects of animal-assisted therapy on anxiety ratings of hospitalized psychiatric patients | 1, 2, 3, 6 |
| 4 | Barker (2003) [37] | Effects of animal-assisted therapy on patients' anxiety, fear, and depression before ECT | 1, 2, 3, 4 |
| 5 | Richeson (2003) [38] | A therapeutic recreation intervention using animal-assisted therapy: effects on the subjective well-being of older adults | 1, 2, 3 |
| 6 | Nathans-Barel (2005) [39] | Animal-assisted therapy ameliorates anhedonia in schizophrenia patients. A controlled pilot study | 1, 2, 3, 4 |
| 7 | Prothmann (2006) [40] | Dogs in child psychotherapy: Effects on state of mind | 1, 2 |
| 8 | Cole (2007) [41] | Animal-assisted therapy in patients hospitalized with heart failure | 6 |
| 9 | Orlandi (2007) [42] | Pet therapy effects on oncological day hospital patients undergoing chemotherapy treatment | 1, 2, 3 |
| 10 | Banks (2008) [43] | Original study: Animal-Assisted Therapy and Loneliness in Nursing Homes: Use of Robotic versus Living Dogs | 2, 3 |
| 11 | Martindale (2008) [44] | Effect of animal-assisted therapy on engagement of rural nursing home resident | 1, 4, 5 |
| 12 | Braun (2009) [45] | Animal-assisted therapy as a pain relief intervention for children | 1, 2, 3 |
| 13 | Chu (2009) [46] | The effect of animal-assisted activity on inpatients with schizophrenia | 2, 3, 5 |
| 14 | Villalta-Gil (2009) [47] | Dog-Assisted Therapy in the Treatment of Chronic Schizophrenia Inpatients | 2, 5, 7 |
| 15 | Moretti (2011) [48] | Pet therapy in elderly patients with mental illness | 1, 5 |
| 16 | Beck (2012) [49] | The effects of animal-assisted therapy on wounded warriors in an Occupational Therapy Life Skills program | 1, 2, 3, 6 |
| 17 | Dietz (2012) [50] | Evaluating animal-assisted therapy in group treatment for child sexual abuse | 1, 2, 3, 4 |
| 18 | Marcus (2012) [51] | Animal-assisted therapy at an outpatient pain management clinic | 1, 2, 3 |
| 19 | Marcus (2013) [52] | Impact of animal-assisted therapy for outpatients with fibromyalgia | 1, 2, 3 |
| 20 | Vrbanac (2013) [53] | Animal assisted therapy and perception of loneliness in geriatric nursing home residents | 1, 2, 3 |
| 21 | Havey (2014) [54] | The effect of animal-assisted therapy on pain medication use after joint replacement | 1, 2, 3 |
| 22 | Nordgren (2014) [55] | Effects of dog-assisted intervention on behavioral and psychological symptoms of dementia | 2, 5 |
| 23 | Barker (2015) [56] | The Effect of an Animal-Assisted Intervention on Anxiety and Pain in Hospitalized Children | 2, 3 |
| 24 | Calcaterra (2015) [57] | Post-operative benefits of animal-assisted therapy in pediatric surgery: a randomized study | 2, 7 |
| 25 | Nurenberg (2015) [58] | Animal-assisted therapy with chronic psychiatric inpatients: equine-assisted psychotherapy and aggressive behavior | 4 |
| Matters causing low quality rating: 1) Lack of randomization, 2) Lack of adequate control group(s), 3) Lack of control for confounders, 4) Insufficiently described experimental design, 5) Underpowered study, 6) Minute treatment effects, 7) High dropout rate. | | | |
